# Characterizing chromatin folding coordinate and landscape with deep learning

**DOI:** 10.1371/journal.pcbi.1008262

**Published:** 2020-09-28

**Authors:** Wen Jun Xie, Yifeng Qi, Bin Zhang

**Affiliations:** Department of Chemistry, Massachusetts Institute of Technology, Cambridge, Massachusetts, United States of America; Rutgers University, UNITED STATES

## Abstract

Genome organization is critical for setting up the spatial environment of gene transcription, and substantial progress has been made towards its high-resolution characterization. The underlying molecular mechanism for its establishment is much less understood. We applied a deep-learning approach, variational autoencoder (VAE), to analyze the fluctuation and heterogeneity of chromatin structures revealed by single-cell imaging and to identify a reaction coordinate for chromatin folding. This coordinate connects the seemingly random structures observed in individual cohesin-depleted cells as intermediate states along a folding pathway that leads to the formation of topologically associating domains (TAD). We showed that folding into wild-type-like structures remain energetically favorable in cohesin-depleted cells, potentially as a result of the phase separation between the two chromatin segments with active and repressive histone marks. The energetic stabilization, however, is not strong enough to overcome the entropic penalty, leading to the formation of only partially folded structures and the disappearance of TADs from contact maps upon averaging. Our study suggests that machine learning techniques, when combined with rigorous statistical mechanical analysis, are powerful tools for analyzing structural ensembles of chromatin.

## Author summary

Chromatin folding, the dynamical process during which chromatin establishes its three-dimensional organization for proper function, is of critical importance. However, it is difficult to visualize and characterize due to challenges associated with live-cell imaging at high temporal and spatial resolution. Here, using a combination of deep learning and statistical mechanical theory, we demonstrate that great insight can be gained into the folding process by analyzing snapshots of chromatin structures taken across a population of cells. Though these static structures are not connected in time, prior research on chemical reactions suggests that fluctuation within the conformational ensemble provides valuable information for uncovering the reaction mechanism. Our analysis reconciles the seemingly contradictory results from different experimental techniques and supports the presence of multiple factors in organizing the chromatin. As single-cell experimental data are becoming routine, the approaches presented here could help with their interpretation to provide more insight into chromatin folding.

## Introduction

Three-dimensional genome organization is expected to play a crucial role in transcription, DNA replication, and repair [1–5]. Significant progress has been made towards its high-resolution characterization as a result of advances in chromosome-conformation-capture based methods such as Hi-C [6, 7]. These methods approximate the 3D distance between pairs of genomic loci using contact frequencies measured via proximity ligation and have revealed many conserved features of genome packaging [8–12]. The emerging picture is a hierarchical organization for interphase chromosomes that ranges from chromatin loops and topologically associating domains (TADs) to compartments at kilobase and megabase scales, respectively [13–15].

Hi-C and related techniques have also provided insight into the dynamical folding process for the establishment of genome organization. In particular, the extrusion model was proposed to explain numerous features of chromatin loops and TADs observed in Hi-C contact maps [16, 17]. It provides a detailed hypothesis on the folding process driven by CCCTC-binding factor (CTCF) and cohesin molecules [18–20]. Several predictions of the extrusion model have been validated with perturbative Hi-C [21–25] and in vitro experiments [26, 27]. Due to its unavoidable ensemble averaging, however, Hi-C cannot capture the heterogeneity within a cell population, and the average picture it presents may be insufficient to uncover the full complexity of genome folding [28, 29].

**Fig 1:**
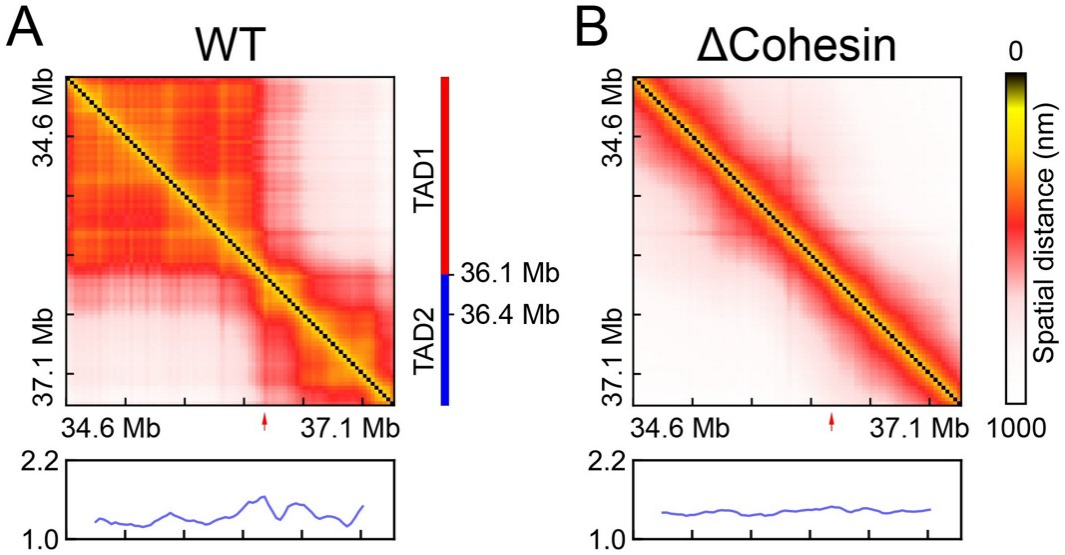
Average distance maps determined using single-cell chromatin structures collected from WT and cohesin-depleted (ΔCohesin) cells. The chromatin segment is Chr21:34.6Mb-37.1Mb of HCT116 cells studied in Ref [34]. Boundary score profiles, whose peaks can be used to identify TAD boundaries and are highlighted with red arrows, are shown below the maps. Detailed definition for the boundary score is provided in the Methods Section. TAD annotation for WT cells is also shown as a guide to the eye.

Many questions on genome folding remain outstanding and necessitate the development of additional experimental techniques and theoretical tools of interpretation. Recently, Zhuang and coworkers applied a super-resolution tracing method [30–34] to characterize single-cell chromatin structures and observed substantial cell-to-cell variation for TAD boundaries [34]. Upon cohesin depletion, in agreement with population Hi-C experiments [25], their study suggested that TADs disappear in ensemble averaged distance matrices (see Fig 1). Remarkably, however, chromatin domains persist in individual cells. The biological implications of these imaging results remain to be explored, and it is unclear what folds the chromatin in cells that lack cohesin molecules and loop extrusion [35]. The large set of single-cell structures provides unprecedented details into chromatin organization but calls for the use of statistical mechanical approaches for its interpretation.

Here we combine deep learning techniques with statistical mechanical tools to investigate the mechanism of genome folding. Specifically, we apply the variational autoencoder (VAE) [36], a deep generative model, to analyze single-cell imaging data and infer a one-dimensional reaction coordinate for chromatin folding. This folding coordinate captures the variation of TAD boundaries in wild-type (WT) configurations and establishes connections among the seemingly random structures in cohesin-depleted cells. It suggests that these structures are intermediate states along the folding pathway to chromatin configurations that bear a striking resemblance to those found in WT cells. Connecting VAE probability of chromatin structures with the free energy cost of folding, we find that the formation of WT-like structures remains energetically favorable even in cohesin-depleted cells. This energetic stabilization leads to partially folded structures with varying domain boundaries observed in single cells. The folding is penalized by the configurational entropy, however, and without the presence of cohesin, chromatin cannot fully commit to the WT-like structures. Our discovery of a weak compartment boundary suggests that phase separation may contribute to chromatin folding in cohesin-depleted cells, and its combination with loop extrusion could underlie the stable and robust TAD formation in WT cells.

## Results

### Deep generative model differentiates chromatin structures from two cell types

In Ref [34], Zhuang and coworkers applied single cell imaging to characterize the organization of a chromatin segment (Chr21:34.6Mb-37.1Mb of HCT116 cells) at high resolution. They found that, in contrary to the average distance map shown in Fig 1, chromatin domains persist upon cohesin removal, an observation that cannot be immediately explained by the loop extrusion model [16, 17]. A detailed analysis of individual chromatin structures from cohesin-depleted cells to reveal their similarity and distinction from WT configurations could provide mechanistic insight into chromatin folding. Such an analysis can be challenging, however, due to the high dimensionality of the data set. Often, it is useful to reduce dimensionality and examine the collective features of the structural ensemble. As demonstrated in prior studies [37–39], focusing on coarsened collective features could facilitate the interpretation of conformational heterogeneity by differentiating functionally meaningful and statistically significant structural fluctuation from random noise.

**Fig 2:**
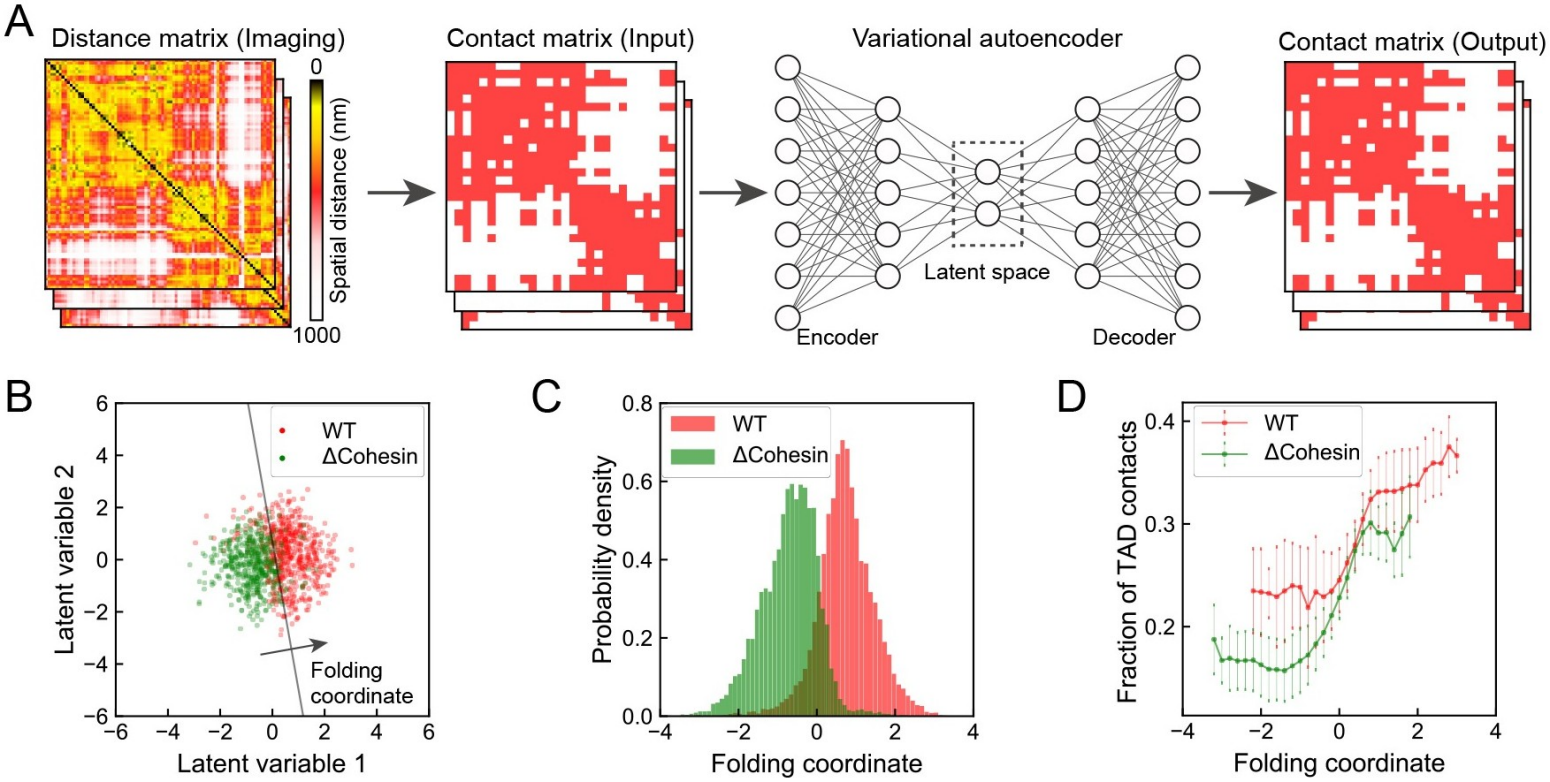
Chromatin folding coordinate derived using deep learning to differentiate chromatin organization in WT and cohesin-depleted cells. (A) Illustration of the variational autoencoder for data processing and low-dimensional embedding. Single-cell chromatin images were first binarized into contact matrices that can be fed into VAE as inputs. The encoder network further projects the high dimensional contacts into a small set of latent variables that best preserve key features of the original data. The decoder network then defines the reconstruction from latent variables to contact matrices. (B) Scatter plot for WT and cohesin-depleted cells in the two-dimensional space of latent variables learned from VAE. The black line represents the decision boundary and the folding coordinate is defined as the distance from the boundary. To avoid overplotting, only 5% of randomly sampled data are shown. For the full dataset, if all the points that fall to the lower left of the boundary were all assigned as cohesin-depleted cells and those on the upper right as WT cells, the misclassification rate is 12.8%. (C) Probability distributions of the folding coordinate for chromatin structures from WT and cohesin-depleted cells. (D) Correlation between the folding coordinate and the fraction of contacts formed within the WT TADs. Error bars represent one standard deviation of uncertainty.

We applied the deep learning framework, VAE, to carry out the dimensionality reduction for an ensemble of chromatin structures from both WT and cohesin-depleted cells. Compared to existing approaches, VAE not only compresses the data into a low-dimensional space with non-linear embedding, but also produces a deep generative model for estimating the statistical probability of each configuration [40–42]. This quantitative aspect is crucial for connecting with thermodynamic analysis discussed in later sections. We converted the 3D positions from single-cell imaging into binarized contact matrices to provide rotationally and translationally invariant representations for chromatin (see Methods Section for details). We then applied VAE over the binarized representations to find two optimal latent variables in an unsupervised manner with an encoder that compresses the contact matrices and a decoder that reconstructs the inputs (Fig 2A).

As shown in Fig 2B, we found an apparent separation between WT (red) and cohesin-depleted (green) cells in the two-dimensional latent space. For the convenience of downstream analysis, from the two latent variables, we further defined a one-dimensional coordinate as the distance from the decision boundary that best separates the two cell types (Fig 2B). We identified the boundary with the support vector machine [43], and WT and cohesin-depleted cells exhibit the largest difference along the direction perpendicular to the boundary. Projecting chromatin configurations onto the folding coordinate leads to a clear separation between the corresponding probability distributions as well (Fig 2C). On the other hand, the two distributions along the direction perpendicular to the folding coordinate (i.e., the direction along the SVM decision boundary) overlap significantly (see S1 Fig). The Kullback-Leibler (KL) divergence that quantifies the distinction between the two one-dimensional probability distributions is 2.1, a value that is comparable to the two-dimensional counterpart (2.0). Therefore, the one-dimensional coordinate is equally effective in differentiating chromatin structures from the two cell types. It is worth pointing out that we processed the same structural ensemble using principal component analysis (PCA) and K-means clustering as well (S2 and S3 Figs. Neither approach separates the two cell types as well as the one-dimensional variable identified here.

The biological significance of the one-dimensional coordinate is evident from its correlation with the fraction of TAD contacts (Fig 2D), which is defined as the ratio between the number of contacts formed inside the two TADs and the total number of contacts. We emphasize that the VAE coordinate was designed to capture the intrinsic variation within the dataset. Its correlation with the fraction of TAD contacts suggests that not only the average difference between the two cell types can be understood from the TAD structure, but the conformational heterogeneity from individual cells is also related to the degree of TAD formation as well.

### Folding coordinate reveals TAD formation in cohesin-depleted cells

To more closely examine the relationship between the VAE coordinate and TAD formation, we characterized the variation of average distance maps along the VAE coordinate. These maps were determined using chromatin structures from either WT or cohesin-depleted cells. The number of cells at various values of the folding coordinate are listed in S1 and S2 Tables.

**Fig 3:**
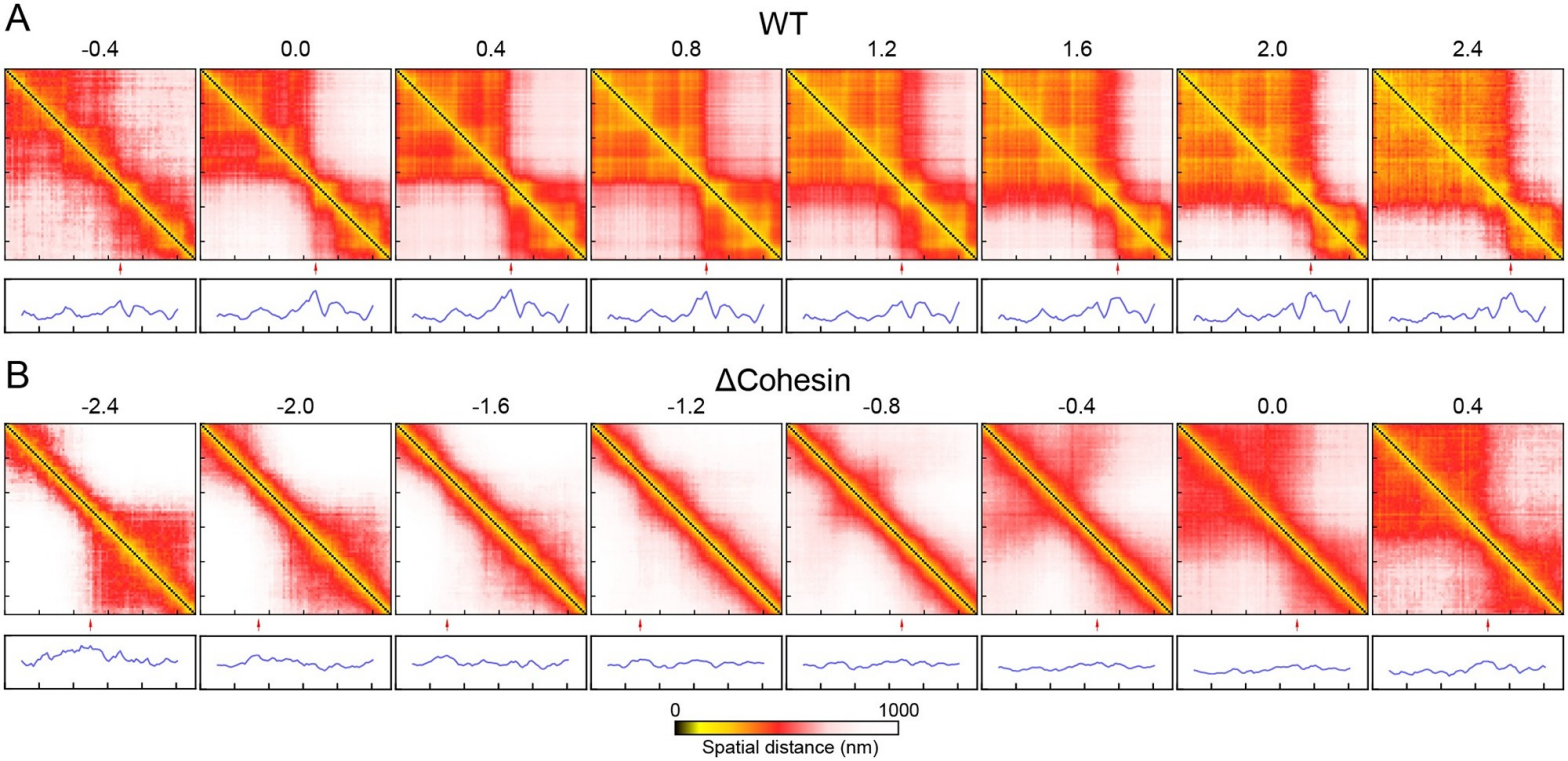
Variation of chromatin distance maps along the folding coordinate for WT and cohesin-depleted cells. Values of the folding coordinate are provided on top of the maps. Boundary score profiles are shown below to highlight the position of TAD boundaries with red arrows.

As shown in Fig 3A, for WT cells, we find that the VAE coordinate captures the heterogeneity of chromatin organization both within a single TAD and across TAD boundaries. For example, chromatin in most cells with the coordinate less than 1.2 exhibits two TADs with a separating boundary at 36.1 Mb. This boundary coincides with the one found in the average distance matrix (Fig 1) and in Hi-C contact map [25]. The contacts within each TAD, however, can vary significantly as the coordinate increases. In particular, the emergence of sub-TADs gives rise to more compact chromatin with decreased spatial distances, and correspondingly, the colormap varies from red to yellow. Interestingly, we also find a significant population of cells, i.e., those with the VAE coordinate larger than 1.2, with a shifted TAD boundary at 36.4 Mb. This chromatin reorganization could alter the regulatory environment for genes (*e.g*., RCAN1 and KCNE1) within this region and may impact their expression profiles.

Remarkably, for cohesin-depleted cells (Fig 3B), variation in distance matrices along the VAE coordinate highlights the gradual formation of chromatin structures with striking resemblance to those found in WT cells. For example, for cells with VAE coordinate values between -1.6 and -0.8, the chromatin segment appears to adopt open, extended configurations and there is no prominent feature in the distance matrices. At large values (≥ 0.0), chromatin adopts two domain-like structures with a boundary identical to that found in WT cells. We note that the observed structural ordering only become apparent after averaging and the conformational ensembles at individual folding coordinates can exhibit substantial heterogeneity (see S4, S5 and S6 Figs).

Close examination of the distance matrices reveals additional subtlety of chromatin folding in cohesin-depleted cells. In particular, though both share similar TAD boundaries, the folded chromatin structures in cohesin-depleted cells are less compact and do not exhibit fine sub-TADs as those from WT cells. In addition, the VAE coordinate also uncovers *off-pathway* configurations at values less than -1.6. In these cells, chromatin exhibits a single domain at the end of the genomic region with a boundary quite different from that of WT cells. This domain must unfold before chromatin can transition into WT-like structures.

The VAE coordinate therefore tracks the degree of foldedness for chromatin and will be referred as the *folding coordinate* in the following. It provides a fresh perspective on the heterogeneity intrinsic to single-cell imaging data [44]. The seemingly random organizations observed in individual cells are, in fact, interrelated to each other as intermediate states along the folding pathway and only differ in the degree of foldedness. What drives the folding transition in cohesin-depleted cells and why doesn’t chromatin from these cells fully commit to the well-folded WT-like structures? In the next two sections, we attempt to address these questions by examining the free energy landscape of chromatin folding and the correlation between structural ordering and energetic stabilization.

### Deep generative model recovers the energy landscape of *in silico* chromatin models

An advantage of VAE is that it provides an estimation for the probability of each chromatin structure represented as a binary contact matrix ***Q***. Such estimations offer a link between the machine learning technique with statistical mechanics since the probability is related to the free energy of contact formation (*F*(***Q***)) via the Boltzmann distribution *P*(***Q***) = *Z*^−1^*e*^−*βF*(***Q***)^, where *Z* is the normalization constant. Before interpreting the folding free energy for chromatin, we first evaluated the accuracy of the VAE probability *P*_VAE_(***Q***) in approximating the actual distribution of molecule conformations, *P*(***Q***).

It is useful to first clarify the physical meaning of *F*(***Q***). Following Wolynes and coworkers [45, 46], we decompose the free energy functional into energetic and entropic contributions

$$\begin{array}{c} F[Q]=U[Q]-TS[Q]. \end{array}$$ (1)

The contact energy *U*(***Q***) accounts for the amount of energy released upon contact formation. *S*(**Q**), on the other hand, corresponds to homogeneous generic properties and describes the general collapse of a polymer chain of length *N*. Therefore, when applied to polymer molecules with different chemical properties but of equal length, the variation in contact free energy will be reflected in *U*(***Q***) while *S*(**Q**) remains the same.

The presence of the entropy term in Eq 1 makes the determination of the free energy functional, and correspondingly the comparison with −log *P*_VAE_(***Q***), difficult. One way to circumvent this challenge is to evaluate the difference of the two quantities from a reference system. In particular,

$$\begin{array}{c} \begin{array}{cc} F\left(Q\right)-F_{\text{ref}}\left(Q\right) & =\left[U\left(Q\right)-U_{\text{ref}}\left(Q\right)\right]-T\left[S\left(Q\right)-S_{\text{ref}}\left(Q\right)\right]\\ & \approx U\left(Q\right)-U_{\text{ref}}\left(Q\right)=\Delta U\left(Q\right). \end{array} \end{array}$$ (2)

The second equation holds if the two polymer systems share similar persistence length and excluded volume effect. In such cases, the microscopic entropic functionals that depends only on generic polymer properties will cancel out. Therefore, if the VAE probability approximates the true distribution well, then the difference between VAE free energies, $-\text{log}[P_{\text{VAE}}\left(Q\right)/P_{\text{VAE}}^{\text{ref}}\left(Q\right)]$, should reproduce Δ*U*(***Q***).

**Fig 4:**
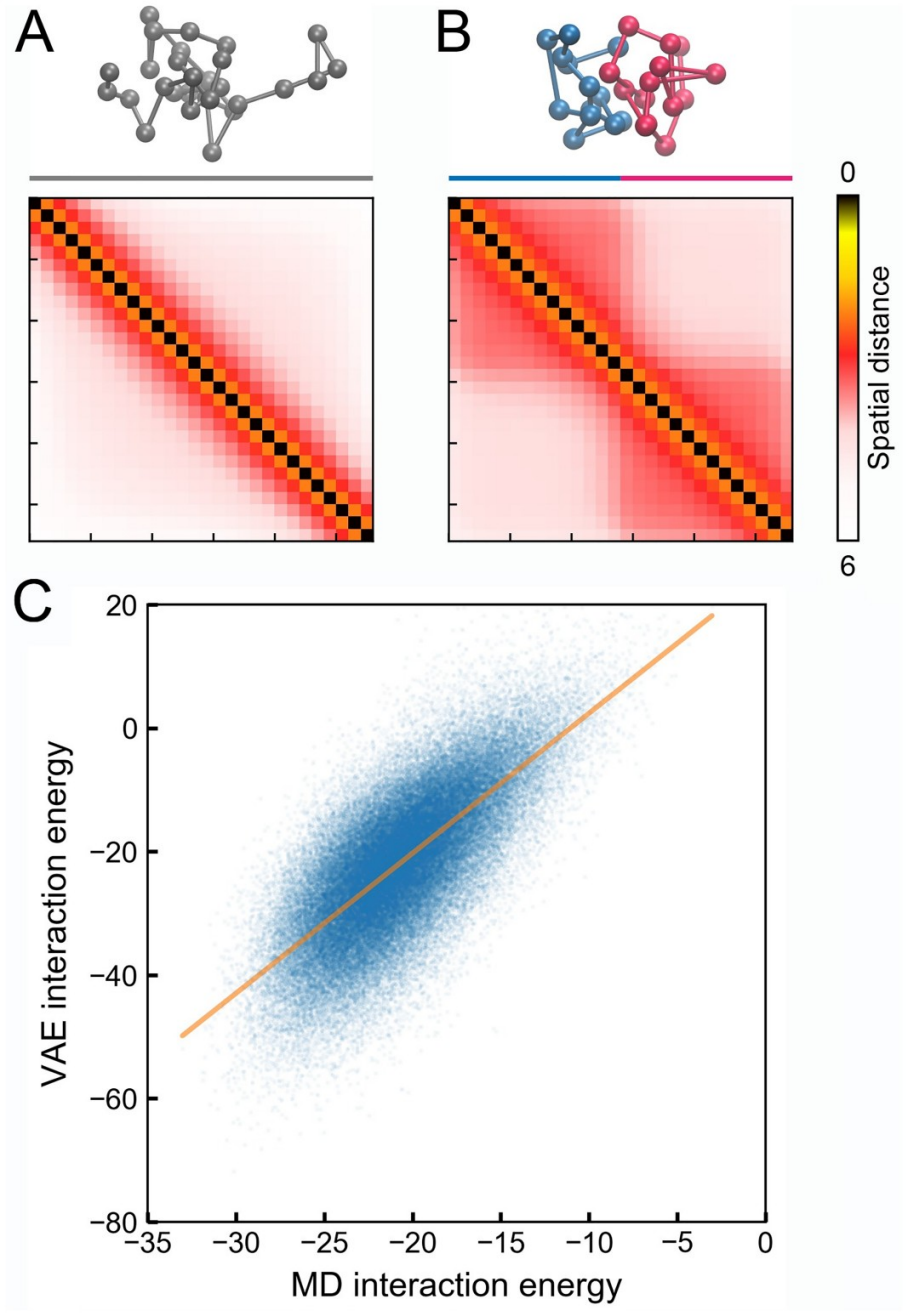
VAE models reproduce the microscopic energy of *in silico* polymer models. (A,B) Representative configurations and average distance matrices for the reference (A) and the chromatin-like (B) polymer. (C) Comparison between the interaction energy calculated from VAE, $-\text{log}[P_{\text{VAE}}\left(Q\right)/P_{\text{VAE}}^{\text{ref}}\left(Q\right)]$, and molecular dynamics simulations, Δ*U*[***Q***]. Energy unit is *k*_B_*T*. The orange line corresponds to a linear fit to the data.

To evaluate its accuracy, we applied VAE to two *in silico* polymer systems for which the contact energy difference of a give molecular conformation can be easily determined. We carried out two computer simulations to collect 3D structures for a reference and a chromatin-like polymer model. The interaction energy in the reference model was fine-tuned to ensure that the average distance between neighboring beads and the overall size of the polymer are comparable to those measured experimentally for chromatin. For the chromatin-like model, in addition to the potential energy defined in the reference system, we introduced attractive interactions for beads within the first and second half of the polymer to promote the formation of domain like structures. Snapshots of the reference and chromatin-like polymers are provided in Fig 4A and 4B, with the simulated average distance matrices shown on the side. Because the two systems share the same basal interactions that define the polymer topology, their entropic functional should be identical.

We then trained two VAE models using a total of 100000 configurations for each polymer. From these two models, we calculated the VAE interaction energy, $-\text{log}[P_{\text{VAE}}\left(Q\right)/P_{\text{VAE}}^{\text{ref}}\left(Q\right)]$, for each one of the chromatin-like configurations. We further determined the corresponding MD interaction energy, Δ*U*[***Q***], by evaluating the potential energy differences in the Cartesian space. As shown in Fig 4C, the two quantities are significantly correlated with each other, with a Pearson correlation coefficient of 0.73 (p-value < 0.001). The slope of the linear fit for the data is slightly larger than 1, with a value of 2.2. This deviation could potentially be a result of the maximization of a lower bound, rather than the true likelihood function in the VAE framework. It is worth mentioning that without removing entropic contributions, the agreement between the VAE free energy, −log *P*_VAE_(***Q***), and the contact energy, *U*(***Q***), is much worse (S7 Fig).

### Balance between enthalpy and entropy dictates TAD formation

Next, we applied VAE over the WT and the cohesin-depleted imaging data separately to derive the corresponding chromatin energy landscapes. We note that these landscapes are deemed effective as chromatin exhibits slow dynamics [47–49] and is subject to perturbations driven by ATP-powered molecular motors [50, 51]. Nevertheless, provided that they can reproduce the corresponding steady-state distributions, effective landscapes are powerful concepts for characterizing non-equilibrium systems [52, 53].

**Fig 5:**
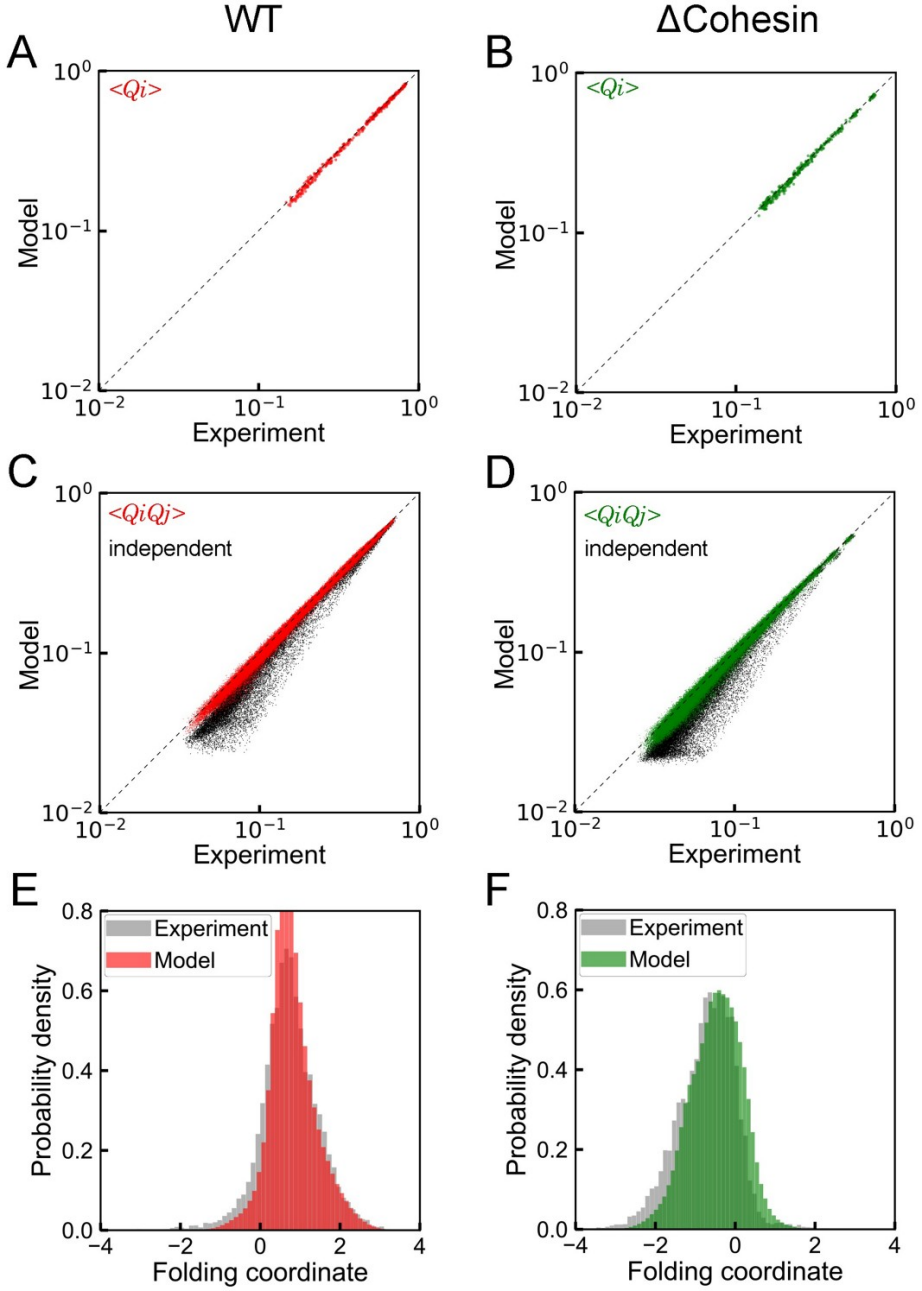
Comparison between experimental value and predictions from VAE. (A, B) Contact probabilities, (C, D) Contact correlations, and (E, F) Probability distributions of the folding coordinate. Parts A, C, and E provide results for WT cells, while parts B, D, and F correspond to the counterparts for cohesin-depleted cells. Estimations for contact correlations based on an independent model are also provided as black dots in parts C and D.

Before analyzing the derived energies, we performed additional tests for the probability distributions estimated by VAE models and evaluated their accuracy in reproducing the measured statistics of chromatin conformation. First, we simulated a total of 10000 chromatin contact matrices by converting randomly distributed latent space variables into contacts using the VAE decoder networks. From these matrices, we computed the average contact frequencies 〈*Q*_*i*_〉 and the pair-wise correlation between contacts 〈*Q*_*i*_ *Q*_*j*_〉. As shown in Fig 5A–5D, values determined from VAE models match well with those from imaging data for both WT and cohesin-depleted cells. It is worth pointing out that a simple independent model fails to capture the cooperativity among chromatin contacts, as evidenced by the deviation between 〈*Q*_*i*_〉 〈*Q*_*j*_〉 and 〈*Q*_*i*_ *Q*_*j*_〉 (Fig 5C and 5D). Finally, we found that VAE models also capture the higher-order collective behavior of chromatin contacts, and the probability distributions of the folding coordinate obtained from simulated contact matrices agree well with the experimental values (Fig 5E and 5F).

**Fig 6:**
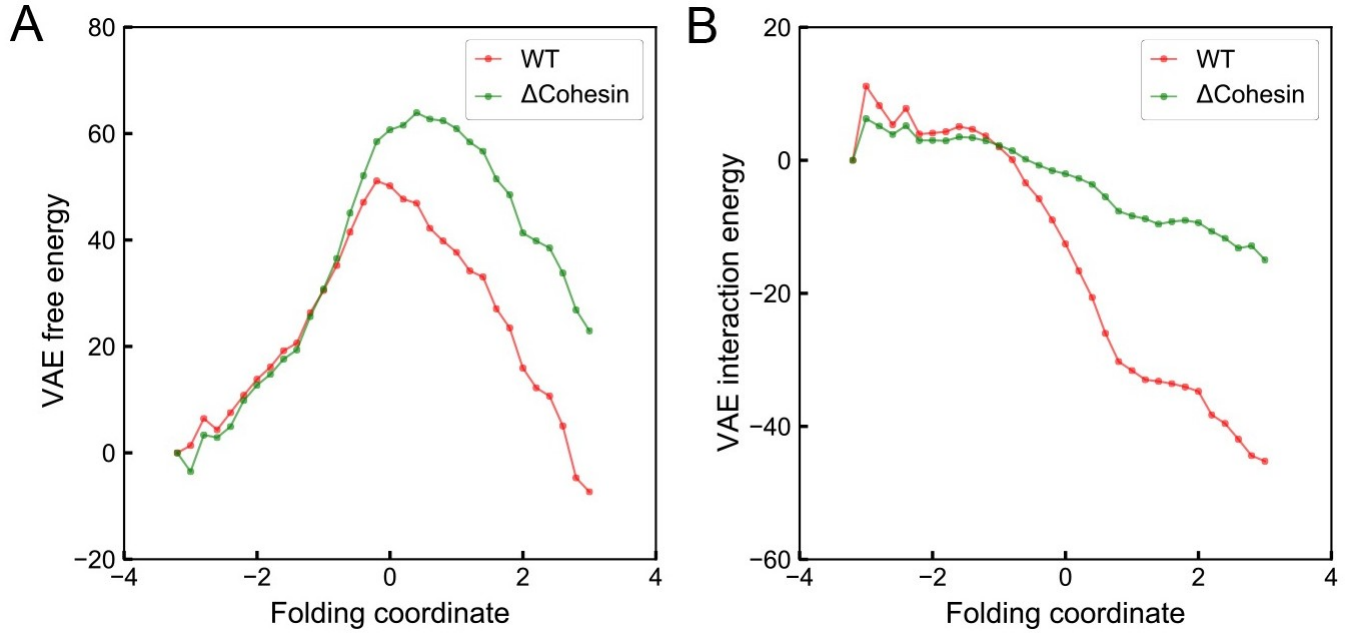
Variation of the free energy (A) and the interaction energy (B) in the unit of *k*_B_*T* along the folding coordinate. The interaction energy is estimated as the free energy difference between the chromatin and a reference polymer as $-\text{log}[P_{\text{VAE}}\left(Q\right)/P_{\text{VAE}}^{\text{ref}}\left(Q\right)]$. See Eq 2 and text for details.

Therefore, both the tests on *in silico* models and the reproducing of experimental data support a quantitative interpretation of the energy landscape inferred from VAE. We next examined the change of various VAE energies along the folding coordinate by averaging the energy over individual chromatin structures from both WT and cohesin-depleted cells. As shown in Fig 6, consistent with the observed low probability of TAD like domains, the free energy, −log[*P*_VAE_(***Q***)], favors unfolded chromatin configurations with negative folding coordinate values for cohesin-depleted cells. However, its difference from the homopolymer free energy introduced in the previous section, $-\text{log}[P_{\text{VAE}}\left(Q\right)/P_{\text{VAE}}^{\text{ref}}\left(Q\right)]$, becomes more negative along the folding coordinate. This quantity, according to Eq 2, measures the strength of specific interactions in chromatin relative to the generic potential of a homopolymer. Since the homopolymer energy itself is weakly attractive and decreases along the folding coordinate (S8 Fig), the specific chromatin interactions favor folded structures even in cohesin-depleted cells. Therefore, the formation of two-domain like structures is indeed energetically stable but must be penalized by the configurational entropy to result in an overall unfavorable free energy. For WT cells, on the other hand, both the free energy and the potential energy stabilizes TADs over unfolded structures.

We note that the free energy, −log[*P*_VAE_(***Q***)], shown in Fig 6 cannot be directly compared with the probability distributions shown in Fig 5. In particular, the mixing entropy that quantifies the number of possible configurations at a given folding coordinate must be accounted for when evaluating the probability of a folding coordinate (see S9 Fig).

## Conclusions and discussion

We applied a state-of-the-art deep learning framework to analyze single-cell imaging data on chromatin organization. By projecting the 3D configurations onto low-dimensional latent variables, we identified a folding coordinate that tracks the progression of TAD formation. Our analysis suggests that the seemingly random structures from individual cohesin-depleted cells can be viewed as intermediate states along the folding transition. Connecting VAE models with the free energy landscape further reconciles the clear intent of folding with the lack of fully commitment. The TAD-like structures remain energetically favorable upon cohesin depletion, driving the formation of chromatin contacts in individual cells. The penalty from the configurational entropy, however, prevents the formation of the full set of contacts to stabilize an entire TAD, resulting in the disappearance of well-defined domains in average distance matrices.

**Fig 7:**
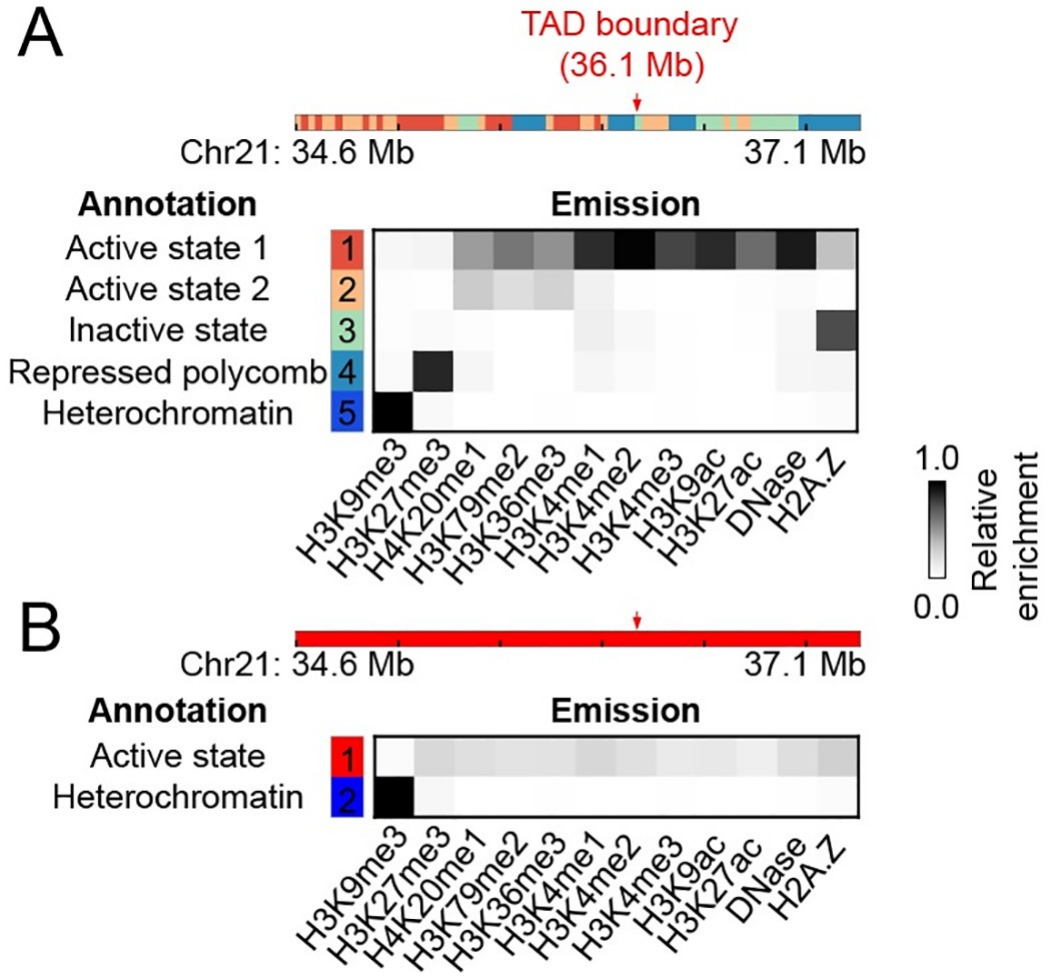
Chromatin state analysis reveals the presence of both active and repressive histone marks in the chromatin region. (A) Results from a five state analysis, with the state assignment shown at the top, and the relative enrichment of various histone marks for each state shown below. The position of the TAD boundary is highlighted with a red arrow. (B) Corresponding results from a two state analysis. The entire chromatin segment is now assigned to an active state.

What are the physicochemical interactions that stabilize the folded WT-like structures in cohesin-depleted cells? We note that the fraction of cohesin-depleted cells with TAD-like structures exceeds 15%, a significant fraction that cannot be explained with residual cohesin molecules that are expected to be much less than 5% after degradation for 6 hours [34, 54]. Numerous studies have demonstrated the importance of phase separation or compartmentalization in genome organization [55–62]. Different regions of the chromatin could adopt distinct post-translational modifications on histone proteins. Such differences, and potentially in combination with the presence of additional intrinsically disordered proteins, could drive the collapse of chromatin into non-overlapping domains in 3D space. An analysis of the underlying combinatorial patterns of twelve histone marks [63] indeed supports this hypothesis. As shown in Fig 7A, the five states defined using the software chromHMM [64] partition the chromatin into active and repress segments at the position corresponding to the TAD boundary. We note that the presence of different chromatin types is not obvious with a coarser classification. As shown in Fig 7B, consistent with the analysis based on Hi-C data [25], this region is assigned as a single active *A* compartment when only two states were used. The presence of both active and repressive histone marks in the chromatin region indicates that phase separation could be driving the partial TAD formation observed in cohesin-depleted cells [59, 60, 65].

Our study reconciles the seemingly contradictory results from population Hi-C experiments and single-cell imaging. Loop extrusion and contributions from a weak compartmentalization boundary, as revealed by the chromatin state analysis, appear to work in harmony to fold the chromatin region studied here.

Because of the complexity of the cell nucleus, the energetic driving forces uncovered in Fig 6 and the corresponding equilibrium interpretation are inherently approximate. Non-equilibrium processes that remodel the chromatin or modify the disordered histone tails could impact chromatin organization and contribute to the thermodynamic quantities extracted from imaging data. A detailed microscopic model of chromatin folding that explicitly considers all the different processes is currently out of reach due to a lack of complete understanding of the various molecular components. In that regard, the approach outlined here is particularly useful as it rigorously accounts for all the contributions in the nucleus while remaining agnostic to the underlying molecular details. As shown in prior studies, such effective equilibrium models can provide accurate descriptions of non-equilibrium steady states in favorable regimes [51, 53, 66].

## Methods

### Imaging data processing

Single-cell super-resolution imaging data were obtained from Ref [34], with a total of 11631 and 9526 chromatin structures for WT and cohesin-depleted cells, respectively. Though the experiments were performed at a 30 kb resolution, we carried out all our analysis at the 90 kb resolution for more accurate estimation of the probability distributions from VAE. We built the distance matrices from 3D positions of every third imaged chromatin segments and converted them into binary contacts with a cutoff of 450 nm. The contact probability between neighboring genomic segments at the 90 kb resolution is about 0.8. For chromatin segments with missing imaging positions, we filled in the corresponding entries in contact matrices with random numbers generated based on the sequence-separation specific average contact probabilities derived from imaging data.

We performed additional tests to confirm that the results shown in Figs 2 and 3 are robust to the cutoff for binarization (S10 Fig) and resolution of the data (S11 Fig).

### Boundary score

To determine the domain boundary in distance maps, we calculated the boundary score profile using the approach introduced by Lazaris and co-workers [67]. For each genomic loci, we first determined their nearest neighbor (X), upstream (U), and downstream (D) regions that are of 180 kb in length. The boundary score is then determined as $\frac{d_{\text{inter}}}{d_{\text{intra}}}$, where *d*_inter_ is the mean distance of all contacts in region X. *d*_intra_ = min(*d*_*U*_, *d*_*D*_) is the minimum average distance of the two neighboring regions.

### Variational autoencoder

We applied VAE both for low dimensional embedding and probability estimation. The imaging data (***Q***) was compressed into the latent variables, ***z***, with an encoding neural network (*q*(***z***|***Q***)). The latent variables were chosen to maximize their potential in representing the original high dimensional data via the optimization of a decoding network (*p*(***Q***|***z***)) to best reconstruct the original imaging data from them.

The probability of a chromatin configuration represented in the binary contact matrix can be formally defined as

$$\begin{array}{c} p(Q)=\int p(Q|z)p(z)dz, \end{array}$$ (3)

where *p*(***z***) is the prior distribution for latent variables. We used the following expression to provide a lower bound on the (log) probability

$$\begin{array}{c} \text{log}\;P_{\text{VAE}}\left(Q\right)≜E_{q}\left[\text{log}\;p\left(Q|z\right)\right]-D_{\text{KL}}\left[q\left(z|Q\right)||p\left(z\right)\right]. \end{array}$$ (4)

The two terms in the above equations correspond to reconstruction error calculated using cross-entropy and the Kullback-Leibler divergence between the posterior and prior distribution of latent variables. We modeled the prior as a multivariate Gaussian distribution [36].

We implemented VAE models in PyTorch [68] and employed the stochastic gradient descent method with the Adam optimizer [69] to derive parameters with a batch size of 500. A total of 1000 epochs with a learning rate of 0.001 was used for model training to ensure the convergence of the loss function. One hidden layer with 200 nodes was used for both the encoding and decoding neural network.

We used different number of latent variables to balance the interpretability and accuracy of the resulting VAE models. The value of the folding coordinate for a given chromatin structure was determined with the two-variable model presented in Fig 2. For this model, the resulting latent variables can be easily visualized and their contribution to distinguishing the two cell types can be gauged straightforwardly. To obtain more accurate probability estimations, we separately trained four VAE models with 25 latent variables for the two set of in silico polymer configurations and the chromatin structures from the two cell types. These models were not used to estimate the folding coordinate, but only for the probability and free energy shown in Figs 4, 5 and 6.

After model training, the probability for observing a chromatin configuration, *P*_VAE_(***Q***), was estimated using Eq 4. A total of 20 independent samples in the latent space was used to ensure convergence when estimating the expectation values.

### Polymer simulations

We carried out two 50 million-step-long polymer simulations using the molecular dynamics package LAMMPS [70]. These simulations were performed with reduced units with *τ*, *σ*, and *ϵ* as the time, length and energy unit, respectively. The timestep was set to *dt* = 0.01*τ*. Langevin dynamics with a damping coefficient of *γ* = 0.5*τ* was used to maintain the temperature at *T* = 1.0. We saved polymer structures at every 500 steps to collect a total of 100000 configurations from each simulation. Simulated polymer configurations were then converted to contact matrices with a cutoff of 3.0*σ* for VAE model parameterization. The cutoff was chosen to ensure that the simulated contact probability between neighboring beads is comparable to the experimental value.

The polymer consists of 28 beads to mimic the 2.5 MB long chromatin region at 90 kb resolution. The energy function for the reference model is defined as

$$\begin{array}{c} U_{\text{ref}}\left(r\right)=U_{\text{b}}\left(r\right)+U_{\text{sc}}\left(r\right)+U_{\text{nb}}\left(r\right). \end{array}$$ (5)

*U*_b_(***r***) is the harmonic bonding potential between neighboring beads with an equilibrium distance of 2.0*σ* and a spring constant of 1.0 *ϵ*/*σ*^2^. *U*_sc_(***r***) is a soft-core potential applied to all the non-bonded pairs to account for the excluded volume effect and to allow for chain crossing [59]. It is equivalent to a capped off Lennard-Jones potential and only incurs a finite energetic cost for overlapping beads. *U*_nb_(***r***) is a weak collapsing potential with the following form

$$\begin{array}{c} U_{\text{nb}}\left(r\right)=\sum_{i,j}\frac{\alpha }{2}[1+\text{tanh}(\eta (r_{\text{c}}-r_{ij}))], \end{array}$$ (6)

where *r*_c_ = 3.0*σ* and *η* = 10.0. *α* = −0.04*ϵ* was chosen such that number of contacts formed by the reference polymer is comparable to that for chromatin. As discussed in the main text, given their equal length and comparable polymer properties, the entropic functional for the in silico polymer should be comparable to that of the real chromatin to ensure the accuracy of Eq 2.

Polymer beads in the chromatin-like model experience additional specific interactions besides those defined in Eq 5. In particular, an attractive potential similar to *U*_nb_(***r***) with *α* = −0.1*ϵ* was applied between beads within the first or second half of the polymer to promote domain formation.

## Supporting information

S1 Fig Probability distributions for chromatin structures from WT and cohesin-depleted cell along the direction perpendicular to the folding coordinate. (i.e., the direction along the SVM decision boundary).
(TIF)

S2 Fig Results from principal component analysis (PCA) of chromatin images. (A) Probability distributions of the first principal component for chromatin structures from WT and cohesin-depleted cells. The KL divergence between the distributions is 1.7. Therefore, compared to the folding coordinate defined in the main text, the principal component performs worse for distinguishing the two cell types. (B,C) Variation of chromatin distance maps along the first principal component for WT (B) and cohesin-depleted cells (C). Values of the first principal component are provided on top of the maps. Boundary score profiles are shown below to highlight the position of TAD boundaries with red arrows. We note that there is a significant difference between the average distance maps from WT and cohesin-depeleted cells at principal component values -1, 1 and 3. These differences indicate that the principal component fails to recognize the distinction among the structures. No such misassignment occurs for the folding coordinate and the average distance maps from two cell types look remarkably similar, as shown in Fig 3 of the main text.
(TIF)

S3 Fig Results from K-means clustering of chromatin images using a total of 10 clusters. (A) Population of individual clusters for WT and cohesin-depleted cells. The overlap ratio between the two cell types is 21.5%. Therefore, compared to the folding coordinate defined in the main text, the k-means clustering performs worse for distinguishing the two cell types. (B,C) Average chromatin distance maps of individual clusters for WT (B) and cohesin-depleted cells (C). Cluster IDs are provided on top of the maps. Boundary score profiles are shown below to highlight the position of TAD boundaries with red arrows. In accord with our main results, we again found that over 15% of cohein-depleted cells (group 2 and 10) exhibit TAD-like chromatin structures. The average distance maps from the most populated groups (1, 4 and 6) are similar to the ones shown in Fig 3 of the main text at various VAE coordinate values as well. Lacking a continuous variable, the physical meaning of the discrete groups and their connection is hard to interpret, however.
(TIF)

S4 Fig Example single-cell distance matrices for WT cells with a folding coordinate of 0.4. (TIF)

S5 Fig Example single-cell distance matrices for cohesin-depleted cells with a folding coordinate of 0.4. (TIF)

S6 Fig Example single-cell distance matrices for cohesin-depleted cells with a folding coordinate of -0.4. (TIF)

S7 Fig Comparison between the VAE free energy (−log *P*_VAE_(Q)) and the potential energy used in molecular dynamics simulations (*U*(Q)) for the chromatin-like polymer (A) and the homopolymer (B). The correlation coefficients between the two energies are -0.32 and -0.15, respectively. Therefore, without removing entropic contributions, the correlation between VAE and MD energy is much worse compared to that shown in Fig 4 of the main text.
(TIF)

S8 FigVariation of the (A) interaction energy and (B) entropy of the reference polymer in the unit of *k*_B_*T* along the folding coordinate.The energies were estimated using the mean number of contacts found in imaged chromatin structures at various folding coordinates. Since the interaction energy for the reference polymer is nearly the same for different folding coordinates, contributions to the free energy change, Δ*F*(***Q***), mainly comes form the entropy, i.e., $\Delta S\left(Q\right)\approx \Delta \text{log}\;P_{\text{VAE}}^{\text{ref}}\left(Q\right)$.(TIF)

S9 FigFree energy after considering mixing entropy and probability distributions of the cells along the folding coordinate.Figs 6A and 5E of the main text represent different quantities and are not supposed to agree with each other. In particular, in Fig 6A, we are plotting $\langle F{\left(Q\right)\rangle }_{q=q_{o}}=-\langle \text{log}{[P_{\text{VAE}}\left(Q\right)\rangle }_{q=q_{o}}.$ The angular brackets ${\langle \cdots \rangle }_{q=q_{o}}$ represent averaging over chromatin structures at a given folding coordinate *q*. This quantity differs from the free energy at the folding coordinate by the mixing entropy, i.e. $F(q_{o})={\langle F(Q)\rangle }_{q=q_{o}}-TS(q_{o}),$ where *T* = 1 is the temperature. The mixing entropy *S*(*q*_*o*_) accounts for the number of possible configurations ***Q*** = {*Q*_*ij*_} at the folding coordinate *q* = *q*_*o*_. Wolynes and coworkers [J. Mol. Biol., 1999, 287:657-674] have introduced an approximate expression the mixing entropy as *S*(*q*_*o*_) = Σ_*ij*_ *Q*_*ij*_(*q*_*o*_) log[*Q*_*ij*_(*q*_*o*_)] + (1 − *Q*_*ij*_(*q*_*o*_)) log[1 − *Q*_*ij*_(*q*_*o*_)]. *Q*_*ij*_(*q*_*o*_) denotes the average contact probability between pairs *i* and *j* computed using all chromatin structures with a folding coordinate of *q*_*o*_. Using the above expression for *S*(*q*_*o*_), we computed *F*(*q*_*o*_) (A) and the corresponding probability distribution $P(q_{o})=\frac{e^{-F(q_{o})}}{\int F(q)dq}$ (B). As shown here, the resulting probability distributions are in good agreement with Fig 5E and 5F. We note that due to the approximate expression for the mixing entropy, an exact match is not expected.(TIF)

S10 Fig Folding coordinate definition is robust to the cutoff used to convert distance matrices into binary contacts for VAE model training. Here we show that the results obtained from processing the imaging data at 90kb resolution with a binarization cutoff of 400 nm are comparable to those shown in Figs 2 and 3 of the main text. (A) Scatter plot for WT and cohesin-depleted (ΔCohesin) cells in the two-dimensional space of latent variables learned from VAE. The black line represents the decision boundary and the folding coordinate is defined as the distance from the boundary. (B) Probability distributions of the folding coordinate for chromatin structures from WT and cohesin-depleted cells. (C) Correlation between the folding coordinate and the fraction of chromatin segments that form contacts within the TADs determined separately using structures from the two cell types. (D,E) Variation of chromatin distance matrices along the folding coordinate for WT (D) and cohesin-depleted cells (E). Values of the folding coordinate are provided on top of the matrices. Boundary score profiles are shown below the maps to highlight TAD boundaries as peaks. Red arrow marks the segment with the largest boundary score.
(TIF)

S11 Fig Folding coordinate definition is robust to the resolution of imaging data used for VAE model training. Here we show that the results obtained from processing the imaging data at 30kb resolution with a binarization cutoff of 300 nm are comparable to those shown in Figs 2 and 3 of the main text. (A) Scatter plot for WT and cohesin-depleted (ΔCohesin) cells in the two-dimensional space of latent variables learned from VAE. The black line represents the decision boundary and the folding coordinate is defined as the distance from the boundary. (B) Probability distributions of the folding coordinate for chromatin structures from WT and cohesin-depleted cells. (C) Correlation between the folding coordinate and the fraction of chromatin segments that form contacts within the TADs determined separately using structures from the two cell types. (D,E) Variation of chromatin distance matrices along the folding coordinate for WT (D) and cohesin-depleted cells (E). Values of the folding coordinate are provided on top of the matrices. Boundary score profiles are shown below the maps to highlight TAD boundaries as peaks. Red arrow marks the segment with the largest boundary score.
(TIF)

S1 Table Number of WT cells at various values of the folding coordinate. (PDF)

S2 Table Number of cohesin-depleted cells at various values of the folding coordinate. (PDF)
