# Supplementary figures and images for: Characterizing chromatin folding coordinate and landscape with deep learning
Source: PLoS Comput Biol. 2020 Sep 28;16(9):e1008262. doi: 10.1371/journal.pcbi.1008262 (PMC7544120; doi:10.1371/journal.pcbi.1008262)

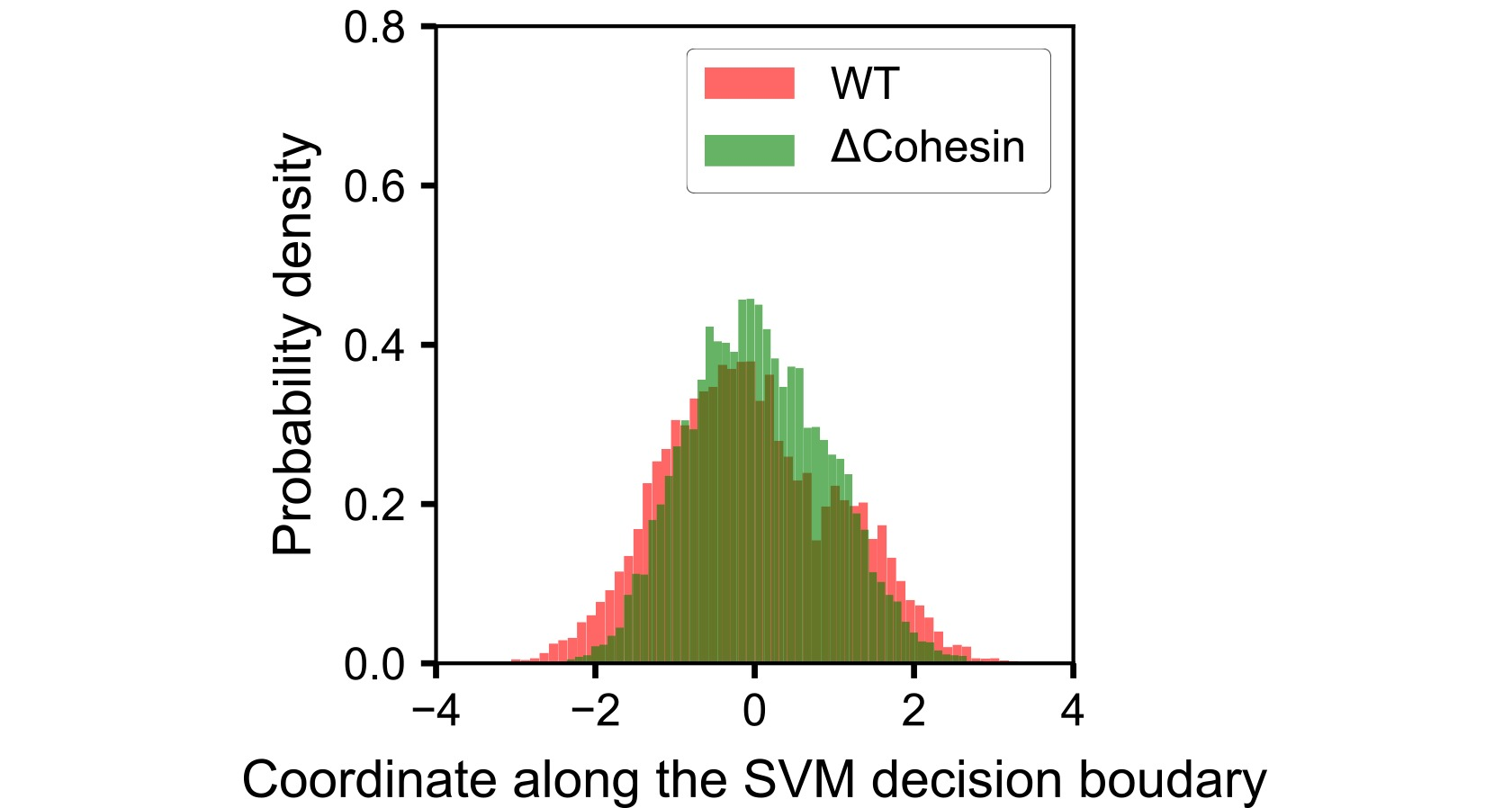

Supplement: S1 Fig — (i.e., the direction along the SVM decision boundary). (TIF) [file pcbi.1008262.s001.tif]

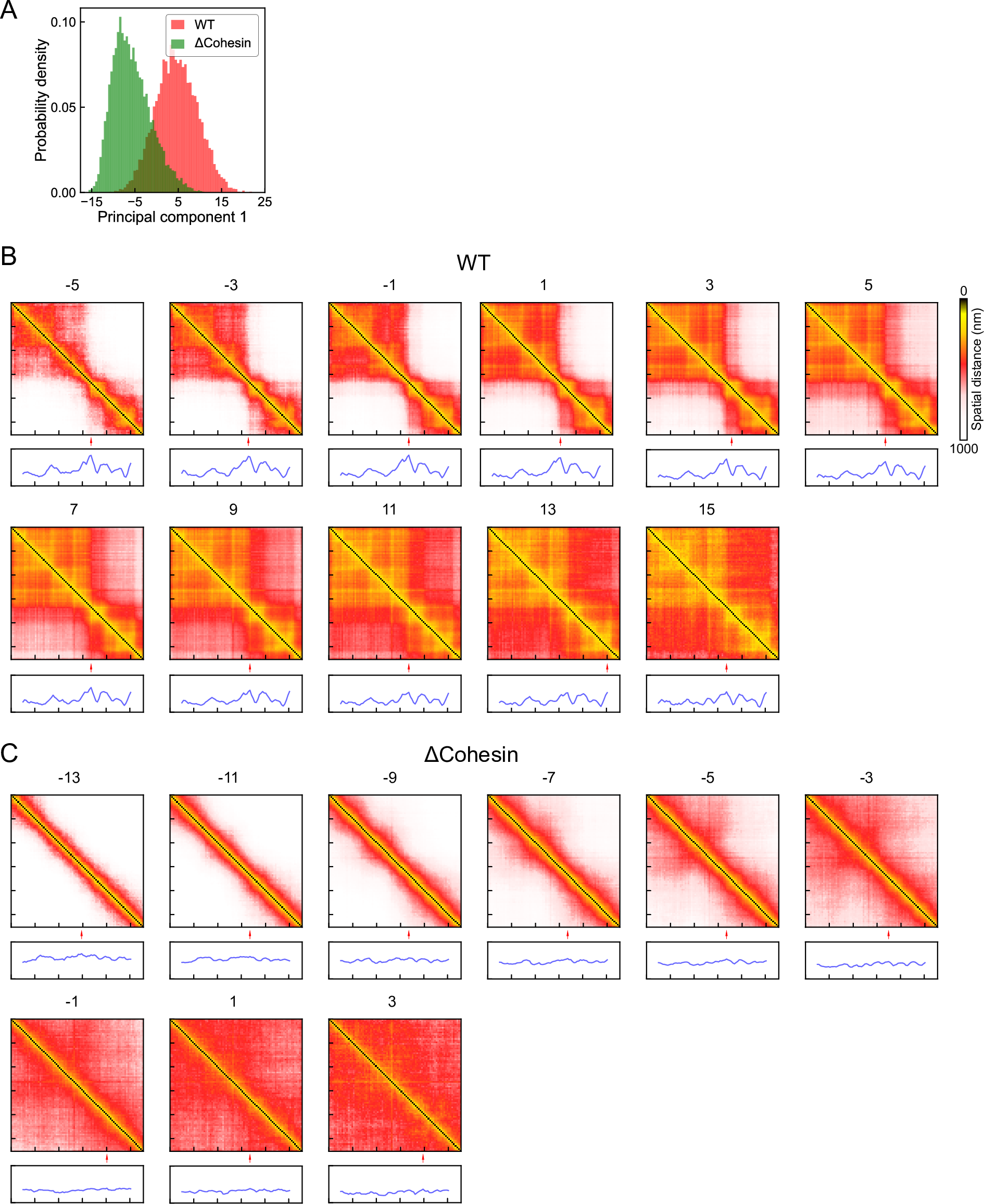

Supplement: S2 Fig — (A) Probability distributions of the first principal component for chromatin structures from WT and cohesin-depleted cells. The KL divergence between the distributions is 1.7. Therefore, compared to the folding coordinate defined in the main text, the principal component performs worse for distinguishing the two cell types. (B,C) Variation of chromatin distance maps along the first principal component for WT (B) and cohesin-depleted cells (C). Values of the first principal component are provided on top of the maps. Boundary score profiles are shown below to highlight the position of TAD boundaries with red arrows. We note that there is a significant difference between the average distance maps from WT and cohesin-depeleted cells at principal component values -1, 1 and 3. These differences indicate that the principal component fails to recognize the distinction among the structures. No such misassignment occurs for the folding coordinate and the average distance maps from two cell types look remarkably similar, as shown in Fig 3 of the main text. (TIF) [file pcbi.1008262.s002.tif]

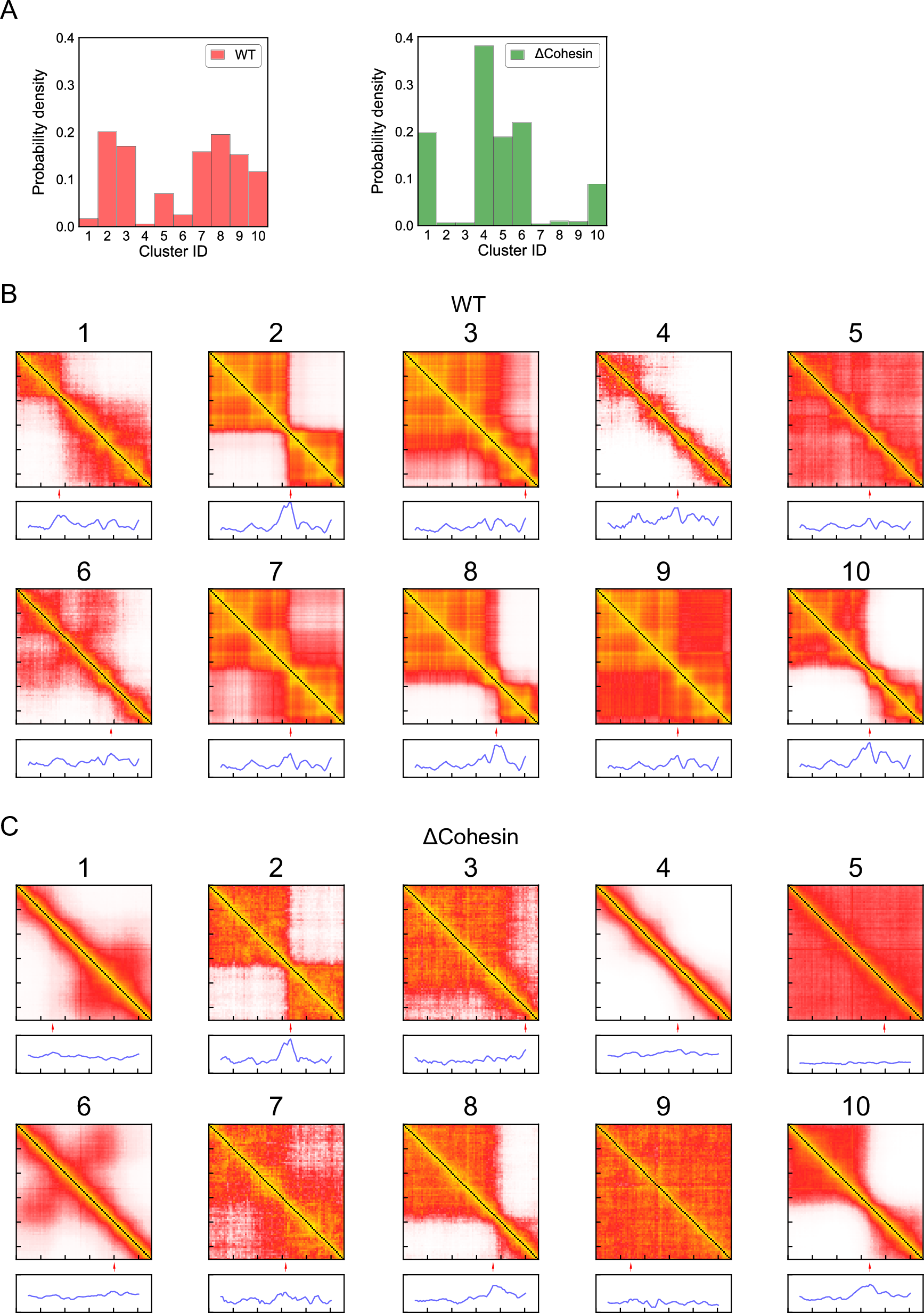

Supplement: S3 Fig — (A) Population of individual clusters for WT and cohesin-depleted cells. The overlap ratio between the two cell types is 21.5%. Therefore, compared to the folding coordinate defined in the main text, the k-means clustering performs worse for distinguishing the two cell types. (B,C) Average chromatin distance maps of individual clusters for WT (B) and cohesin-depleted cells (C). Cluster IDs are provided on top of the maps. Boundary score profiles are shown below to highlight the position of TAD boundaries with red arrows. In accord with our main results, we again found that over 15% of cohein-depleted cells (group 2 and 10) exhibit TAD-like chromatin structures. The average distance maps from the most populated groups (1, 4 and 6) are similar to the ones shown in Fig 3 of the main text at various VAE coordinate values as well. Lacking a continuous variable, the physical meaning of the discrete groups and their connection is hard to interpret, however. (TIF) [file pcbi.1008262.s003.tif]

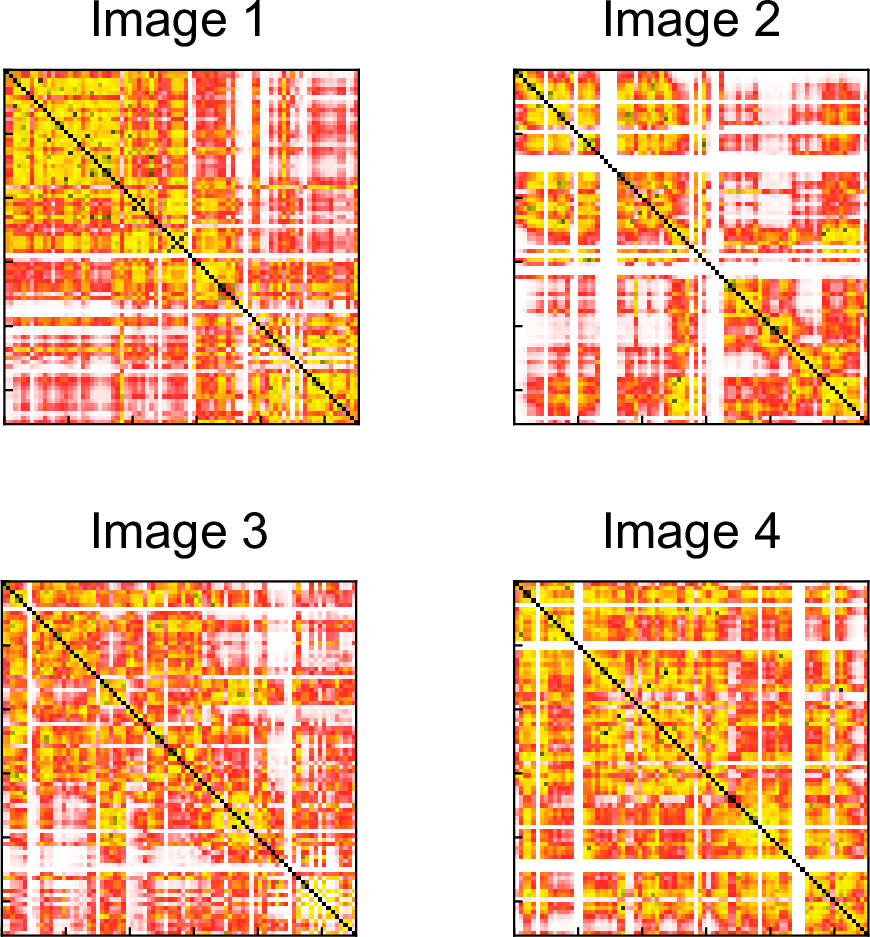

Supplement: S4 Fig — (TIF) [file pcbi.1008262.s004.tif]

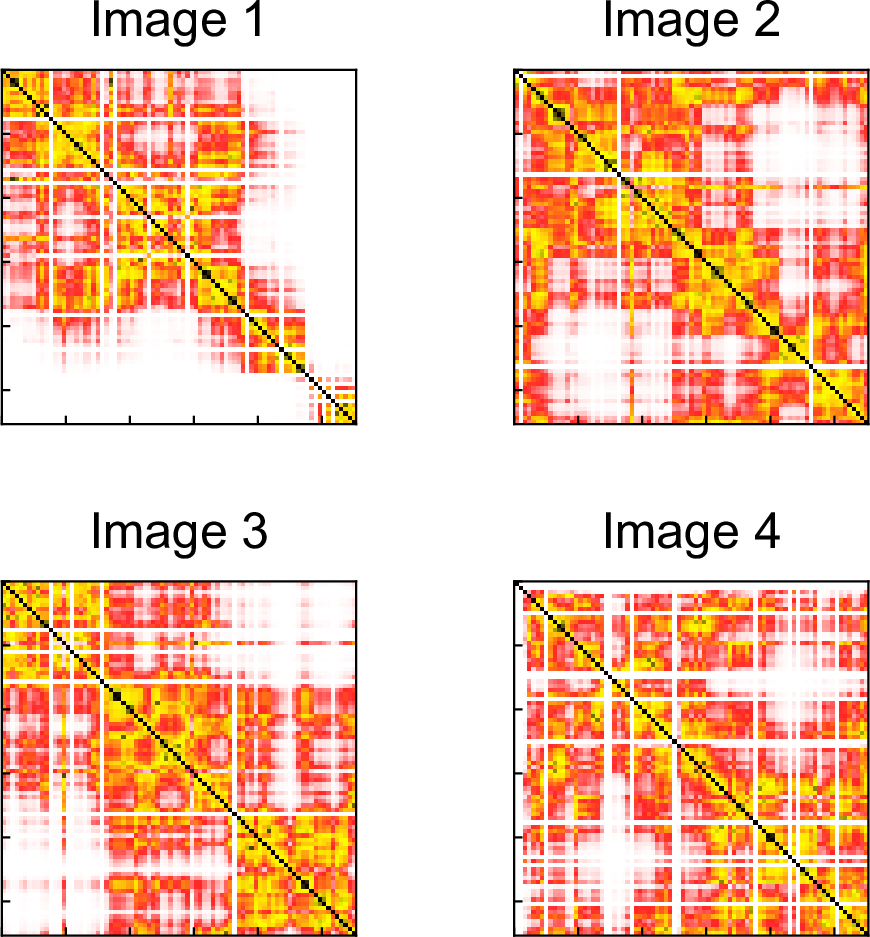

Supplement: S5 Fig — (TIF) [file pcbi.1008262.s005.tif]

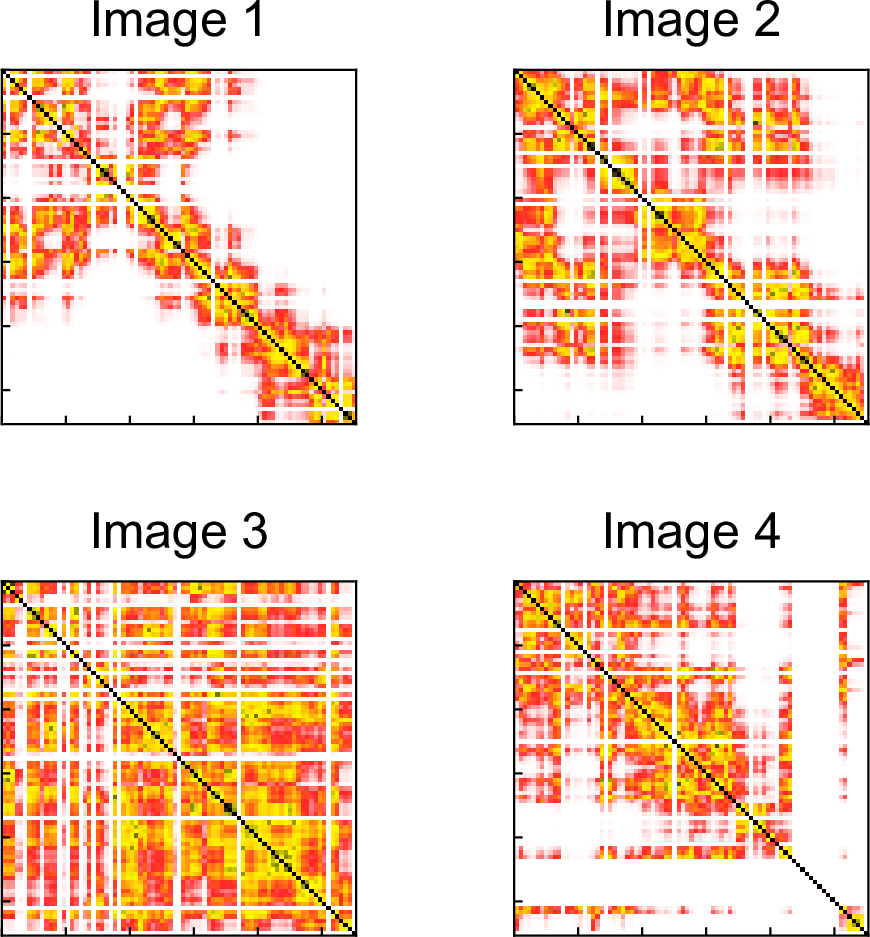

Supplement: S6 Fig — (TIF) [file pcbi.1008262.s006.tif]

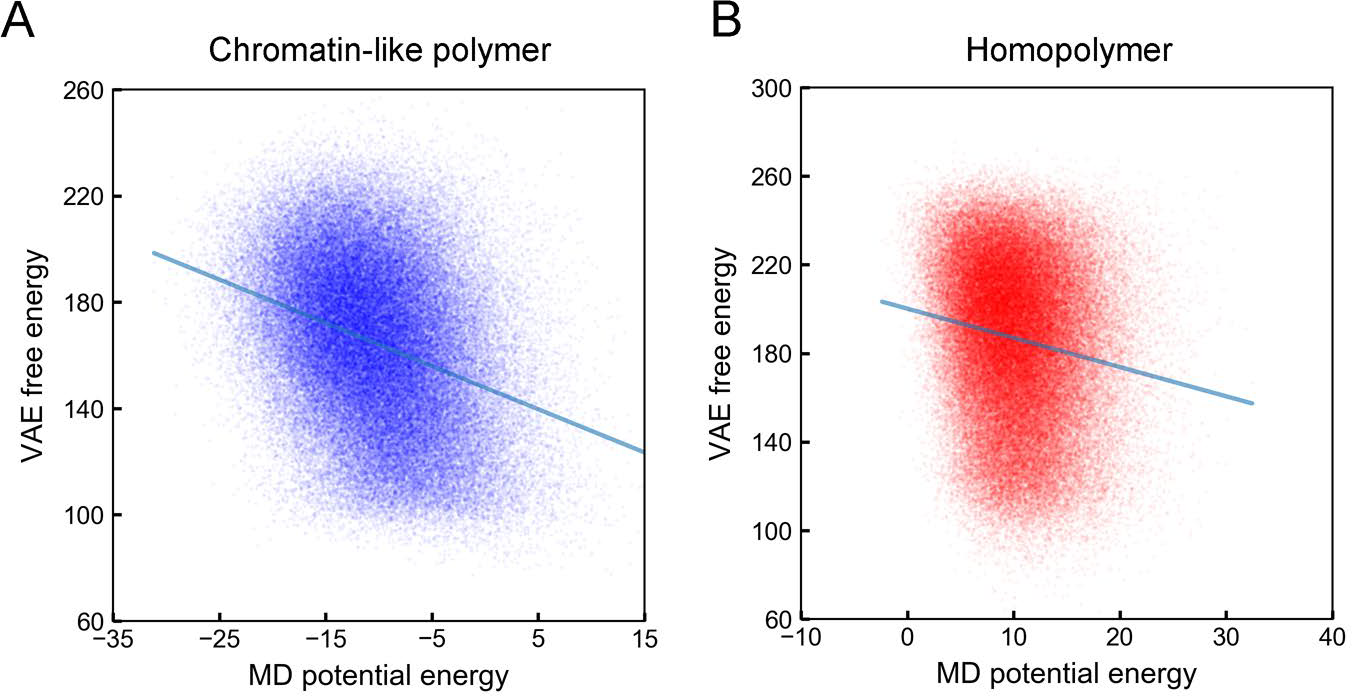

Supplement: S7 Fig — The correlation coefficients between the two energies are -0.32 and -0.15, respectively. Therefore, without removing entropic contributions, the correlation between VAE and MD energy is much worse compared to that shown in Fig 4 of the main text. (TIF) [file pcbi.1008262.s007.tif]

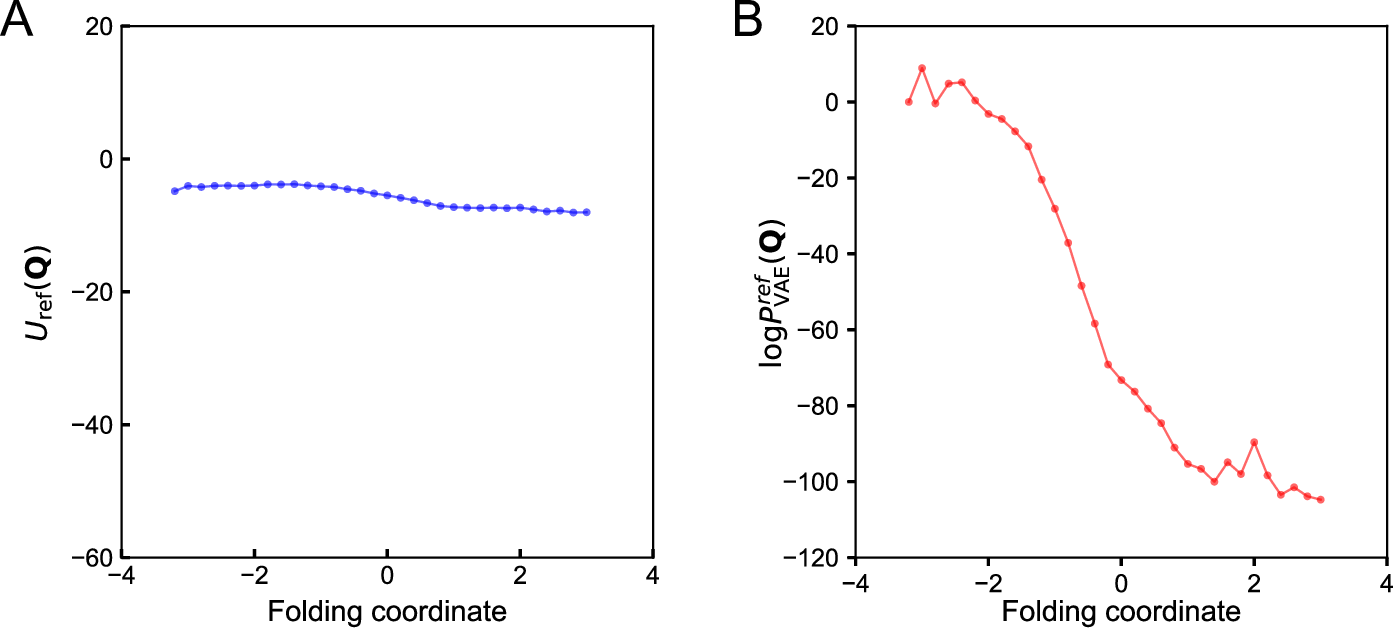

Supplement: S8 Fig — The energies were estimated using the mean number of contacts found in imaged chromatin structures at various folding coordinates. Since the interaction energy for the reference polymer is nearly the same for different folding coordinates, contributions to the free energy change, ΔF(Q), mainly comes form the entropy, i.e., ΔS(Q)≈ΔlogPVAEref(Q). (TIF) [file pcbi.1008262.s008.tif]

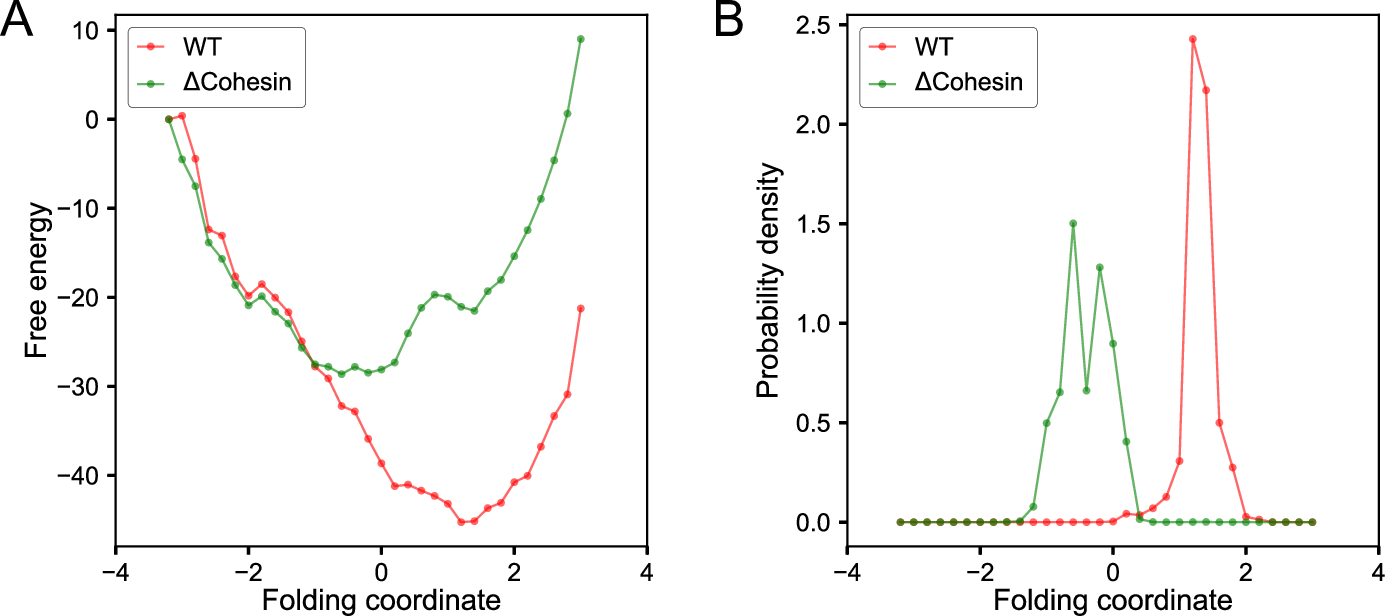

Supplement: S9 Fig — Figs 6A and 5E of the main text represent different quantities and are not supposed to agree with each other. In particular, in Fig 6A, we are plotting 〈F(Q)〉q=qo=-〈log[PVAE(Q)〉q=qo. The angular brackets 〈⋯〉q=qo represent averaging over chromatin structures at a given folding coordinate q. This quantity differs from the free energy at the folding coordinate by the mixing entropy, i.e. F(qo)=〈F(Q)〉q=qo−TS(qo), where T = 1 is the temperature. The mixing entropy S(qo) accounts for the number of possible configurations Q = {Qij} at the folding coordinate q = qo. Wolynes and coworkers [J. Mol. Biol., 1999, 287:657-674] have introduced an approximate expression the mixing entropy as S(qo) = Σij Qij(qo) log[Qij(qo)] + (1 − Qij(qo)) log[1 − Qij(qo)]. Qij(qo) denotes the average contact probability between pairs i and j computed using all chromatin structures with a folding coordinate of qo. Using the above expression for S(qo), we computed F(qo) (A) and the corresponding probability distribution P(qo)=e−F(qo)∫F(q)dq (B). As shown here, the resulting probability distributions are in good agreement with Fig 5E and 5F. We note that due to the approximate expression for the mixing entropy, an exact match is not expected. (TIF) [file pcbi.1008262.s009.tif]

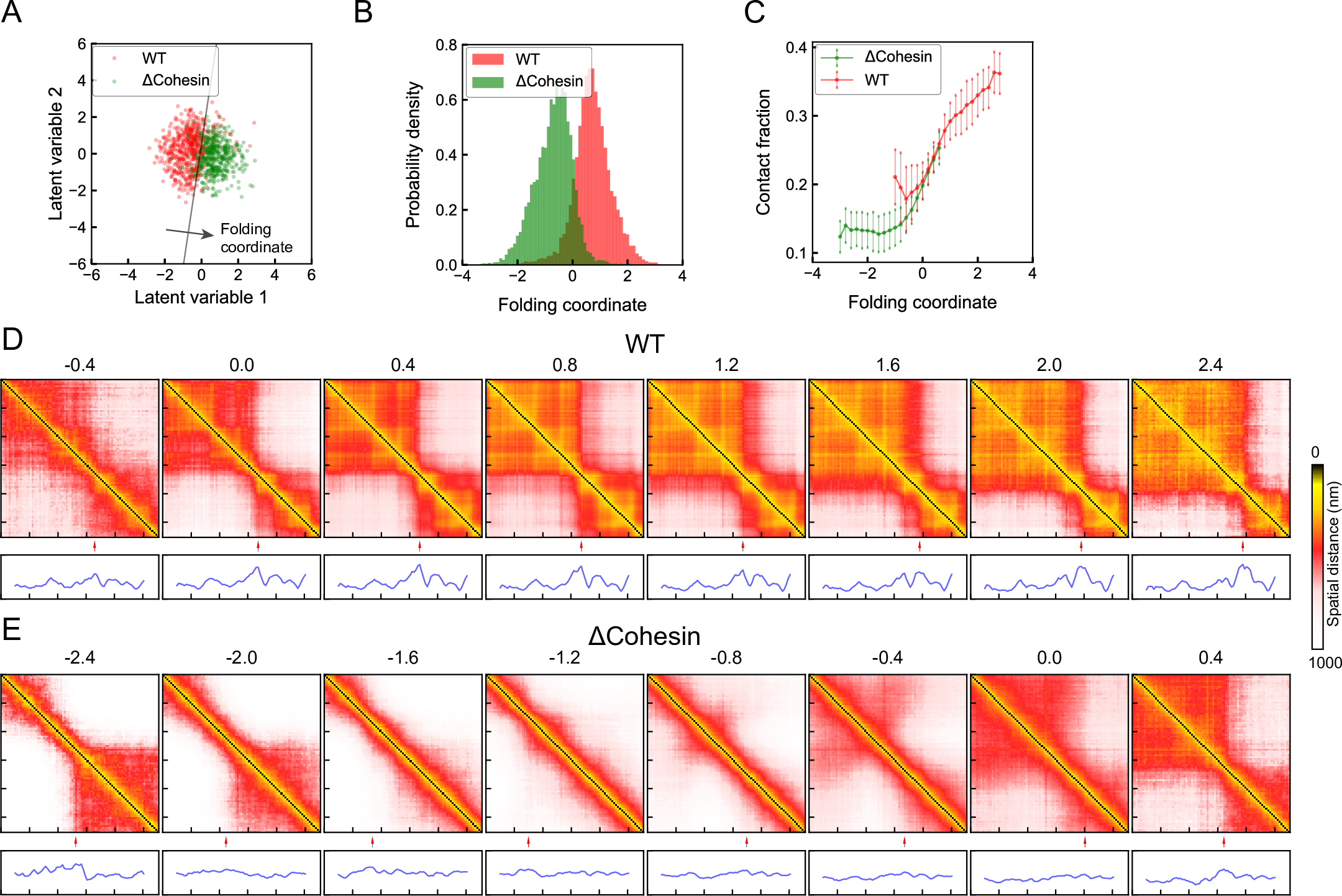

Supplement: S10 Fig — Here we show that the results obtained from processing the imaging data at 90kb resolution with a binarization cutoff of 400 nm are comparable to those shown in Figs 2 and 3 of the main text. (A) Scatter plot for WT and cohesin-depleted (ΔCohesin) cells in the two-dimensional space of latent variables learned from VAE. The black line represents the decision boundary and the folding coordinate is defined as the distance from the boundary. (B) Probability distributions of the folding coordinate for chromatin structures from WT and cohesin-depleted cells. (C) Correlation between the folding coordinate and the fraction of chromatin segments that form contacts within the TADs determined separately using structures from the two cell types. (D,E) Variation of chromatin distance matrices along the folding coordinate for WT (D) and cohesin-depleted cells (E). Values of the folding coordinate are provided on top of the matrices. Boundary score profiles are shown below the maps to highlight TAD boundaries as peaks. Red arrow marks the segment with the largest boundary score. (TIF) [file pcbi.1008262.s010.tif]

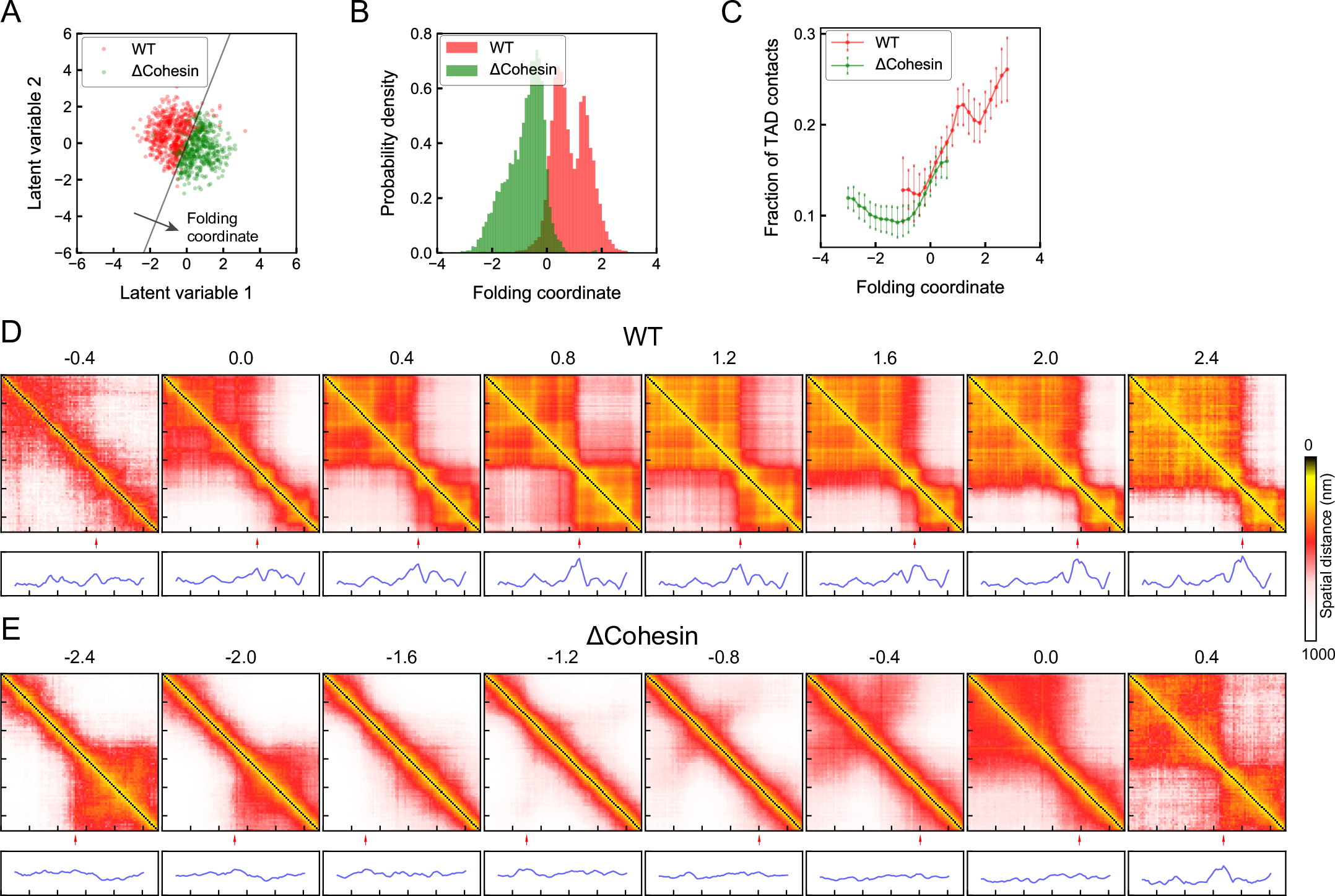

Supplement: S11 Fig — Here we show that the results obtained from processing the imaging data at 30kb resolution with a binarization cutoff of 300 nm are comparable to those shown in Figs 2 and 3 of the main text. (A) Scatter plot for WT and cohesin-depleted (ΔCohesin) cells in the two-dimensional space of latent variables learned from VAE. The black line represents the decision boundary and the folding coordinate is defined as the distance from the boundary. (B) Probability distributions of the folding coordinate for chromatin structures from WT and cohesin-depleted cells. (C) Correlation between the folding coordinate and the fraction of chromatin segments that form contacts within the TADs determined separately using structures from the two cell types. (D,E) Variation of chromatin distance matrices along the folding coordinate for WT (D) and cohesin-depleted cells (E). Values of the folding coordinate are provided on top of the matrices. Boundary score profiles are shown below the maps to highlight TAD boundaries as peaks. Red arrow marks the segment with the largest boundary score. (TIF) [file pcbi.1008262.s011.tif]
