# Supplementary material for: Characterizing chromatin folding coordinate and landscape with deep learning
Source: PLoS Comput Biol. 2020 Sep 28;16(9):e1008262. doi: 10.1371/journal.pcbi.1008262 (PMC7544120; doi:10.1371/journal.pcbi.1008262)
Supplement: S1 Table — (PDF) [file pcbi.1008262.s012.pdf]

Table S1. Number of WT cells at various values of the folding coordinate.

Folding coordinate	Cell count
-3.4	1
-3.2	2
-3.0	4
-2.8	4
-2.6	3
-2.4	5
-2.2	12
-2.0	19
-1.8	15
-1.6	30
-1.4	31
-1.2	45
-1.0	64
-0.8	105
-0.6	157
-0.4	222
-0.2	370
0.0	643
0.2	1014
0.4	1298
0.6	1591
0.8	1580
1.0	1162
1.2	908
1.4	766
1.6	547
1.8	402
2.0	236
2.2	159
2.4	106
2.6	66
2.8	33
3.0	20
3.2	4
3.4	4
3.6	1
3.8	2
