# Supplementary material for: Characterizing chromatin folding coordinate and landscape with deep learning
Source: PLoS Comput Biol. 2020 Sep 28;16(9):e1008262. doi: 10.1371/journal.pcbi.1008262 (PMC7544120; doi:10.1371/journal.pcbi.1008262)
Supplement: S2 Table — (PDF) [file pcbi.1008262.s013.pdf]

**Table S2.** Number of cohesin-depleted cells at various values of the folding coordinate.

| Folding coordinate | Cell count |
|---|---|
| -3.8 | 2 |
| -3.6 | 1 |
| -3.4 | 8 |
| -3.2 | 10 |
| -3.0 | 24 |
| -2.8 | 24 |
| -2.6 | 58 |
| -2.4 | 100 |
| -2.2 | 153 |
| -2.0 | 206 |
| -1.8 | 330 |
| -1.6 | 441 |
| -1.4 | 589 |
| -1.2 | 639 |
| -1.0 | 799 |
| -0.8 | 1061 |
| -0.6 | 1100 |
| -0.4 | 1119 |
| -0.2 | 1053 |
| 0.0 | 850 |
| 0.2 | 482 |
| 0.4 | 234 |
| 0.6 | 90 |
| 0.8 | 33 |
| 1.0 | 28 |
| 1.2 | 28 |
| 1.4 | 20 |
| 1.6 | 18 |
| 1.8 | 12 |
| 2.0 | 5 |
| 2.2 | 6 |
| 2.4 | 1 |
| 2.8 | 2 |
